# Cell Death-Autophagy Loop and Glutamate-Glutamine Cycle in Amyotrophic Lateral Sclerosis

**DOI:** 10.3389/fnmol.2017.00231

**Published:** 2017-07-21

**Authors:** Shu Yuan, Zhong-Wei Zhang, Zi-Lin Li

**Affiliations:** ^1^College of Resources, Sichuan Agricultural University Chengdu, China; ^2^Department of Cardiovascular Surgery, General Hospital of Lanzhou Military Region Lanzhou, China

**Keywords:** amyotrophic lateral sclerosis, cell death—incomplete autophagy loop, glutamate-glutamine cycle, trehalose, Ebselen

## Abstract

Although we know that amyotrophic lateral sclerosis (ALS) is correlated with the glutamate-mediated corticomotor neuronal hyperexcitability, detailed ALS pathology remains largely unexplained. While a number of drugs have been developed, no cure exists so far. Here, we propose a hypothesis of neuronal cell death—incomplete autophagy positive-feedback loop—and summarize the role of the neuron-astrocyte glutamate-glutamine cycle in ALS. The disruption of these two cycles might ideally retard ALS progression. Cerebrovascular injuries (such as multiple embolization sessions and strokes) induce neuronal cell death and the subsequent autophagy. ALS impairs autophagosome-lysosome fusion and leads to magnified cell death. Trehalose rescues this impaired fusion step, significantly delaying the onset of the disease, although it does not affect the duration of the disease. Therefore, trehalose might be a prophylactic drug for ALS. Given that a major part of neuronal glutamate is converted from glutamine through neuronal glutaminase (GA), GA inhibitors may decrease the neuronal glutamate accumulation, and, therefore, might be therapeutic ALS drugs. Of these, Ebselen is the most promising one with strong antioxidant properties.

## Introduction

Amyotrophic lateral sclerosis (ALS) symptoms are characterized by concomitant upper and lower motor neuron signs, with inexorable disease progression (Geevasinga et al., 2016). Typically, ALS patients exhibit a focal onset (limb or bulbar region) and development in an apparently active manner involving other body regions, such as global muscle wasting and weakness, with respiratory muscle dysfunction at the terminal stage of the disease (Geevasinga et al., 2016). About 50% of all ALS patients die within 3 years after the onset of symptoms, and 90% of ALS patients die within 5 years (Kiernan et al., 2011). Atypical ALS phenotypes are characterized by either merely upper motor neuron dysfunction (named primary lateral sclerosis, PLS) or lower motor neuronal signs encompassing flail leg and flail arm syndromes (named progressive muscular atrophy, PMA; Kiernan et al., 2011).

Eisen et al. (1992) proposed the primacy of corticomotor neurons in what has come to be known as the dying-forward hypothesis of ALS pathogenesis, which states that ALS begins centrally, with anterior horn cell degeneration mediated by corticomotor neuronal hyperexcitability via the trans-synaptic glutamate-mediated excitotoxic processes. In contrast, other two concepts of cortical dysfunction have also been put forward. The dying-back theory proposes that the disease is mainly a lower motor neuron disorder that is caused by pathogens transported from neuromuscular junctions to cell bodies in a retrograde manner (Williamson and Cleveland, 1999; Fischer et al., 2004). The independent-degeneration theory presumes that upper motor and lower motor neurons degenerate concurrently but independently, may be in a stochastic manner (Ravits et al., 2007). In particular, cortical dysfunction has been identified as an intrinsic feature of most ALS cases, suggesting that the dying-forward mechanism is the most important in the atypical ALS phenotypes (Vucic et al., 2013b).

Recent clinical, physiological, neurological genetic and biochemical advances have uncovered the importance of cortical hyperexcitability as a pathological biomarker in ALS (Eisen et al., 1992; Eisen and Weber, 2001; Geevasinga et al., 2016). Glutamate is the main excitatory neurotransmitter in the central nervous system. During axonal depolarization, glutamate is released from presynaptic neurons and it activates specific metabotropic and ionotropic glutamate receptors located on the postsynaptic neuron membrane (Heath and Shaw, 2002). The excitatory signal is terminated by the removal of glutamate from the synaptic cleft by glutamate re-uptake transporters. Excessive activation of the glutamate receptors results in the increased influx of Na^+^ and Ca^2+^ ions causing excitotoxicity (Heath and Shaw, 2002; Geevasinga et al., 2016). Trans-synaptic excessive glutamate-induced motor neuron degeneration is the key mechanism; however, the exact causes of ALS, especially its relationships to other diseases, are still largely unknown.

In 2016, Riluzole, an anti-glutamatergic agent, was the only Food and Drug Administration-approved drug for ALS (Bensimon et al., 1994; Lacomblez et al., 1996; Vucic et al., 2013a). However, Riluzole can only slow down the progression of ALS; the drug cannot stop it (Vucic et al., 2013a).

## The Role of Autophagy in ALS

A previous study has suggested that multiple embolization sessions of cerebral arteriovenous malformation (AVM; especially with significant perinidal angiogenesis) may contribute to the subsequent development of ALS (Valavanis et al., 2014). A further study confirmed this association, albeit small in absolute incidence, between AVM and ALS, with additional significant correlations between ALS and prior strokes (either hemorrhagic or ischemic) or transient ischemic attack (Lacorte et al., 2016; Turner et al., 2016). Therefore, cerebrovascular injury from a variety of causes, including embolization procedures for AVM, may be a risk factor for ALS (Turner et al., 2016).

Cerebrovascular injuries usually induce neuronal cell death and autophagy occurring at adjacent cells (Fu et al., 2016). For example, embolization sessions to the patients with rare AVM architecture characterized by significant perinidal angiogenesis may cause ischemia of the perinidal brain parenchyma (Valavanis et al., 2014) and presumably the subsequent cell death and autophagy.

Many studies in *in vitro* cell lines and animal models of ALS demonstrated enhanced autophagic activity in the disease (Nassif and Hetz, 2011; Zhang et al., 2011, 2014). The disease is accompanied by the occurrence of autophagy-mediated clearance of mutant superoxide dismutase 1 (SOD1) and TDP43 proteins, the latter of which is another ALS marker protein (Ling et al., 2015).

In normal conditions, autophagy shows a protective role in neuronal cell survival by removing damaged proteins (Fu et al., 2016; Tang et al., 2016). Hetz et al. (2009) indicated that a deficiency in X-box-binding protein-1 (XBP-1; an unfolded protein response transcription factor) increased autophagy levels in the central nervous system that correlated with enhanced SOD1 autophagic degradation, and resulted in a significant delay in ALS progression. Progesterone activates autophagy and exerts neuroprotective effects for brain ischemia, traumatic brain injury, spinal cord injury and ALS (Kim et al., 2013). Inhibition of autophagy by 3-methyladenine reversed the neuroprotective effects of progesterone (Kim et al., 2013). The PI-3-kinase/Akt kinase inhibitor wortmannin also reduces the neuroprotective effects of angiogenin in primary motor neuron cultures via inhibiting autophagy (Kieran et al., 2008). Furthermore, chloroquine represses lysosomal degradation through neutralizing lysosomal acidic pH, which is required for the activation of autophagic degradation (Vakifahmetoglu-Norberg et al., 2015). Apparent neuronal cell death was observed in chloroquine-treated mice (Dai et al., 2010).

Autophagy activators do not always show beneficial effects on ALS progression. Rapamycin activates autophagy by inhibiting the mammalian target of rapamycin (mTOR) kinase, and it has been shown to have protective effects in several mouse models of some neurodegenerative diseases (Berger et al., 2006; Malagelada et al., 2010; Spilman et al., 2010). However, rapamycin may exaggerate motor neuron loss and exacerbate disease progression in the SOD1^G93A^ ALS-model mouse (Zhang et al., 2011). The rapamycin-treated ALS model mice had a significantly shorter time period from disease onset to death (Zhang et al., 2011). This study suggests that the autophagy pathway may not only operate as a cleaning-up mechanism. Augmenting autophagy levels above a certain threshold may lead to detrimental effects in neuronal function and survival (Nassif and Hetz, 2011; Zhang et al., 2014).

## Trehalose Rescues Impaired Lysosomal Fusion and Improves the ALS Course

**Figure 1:**
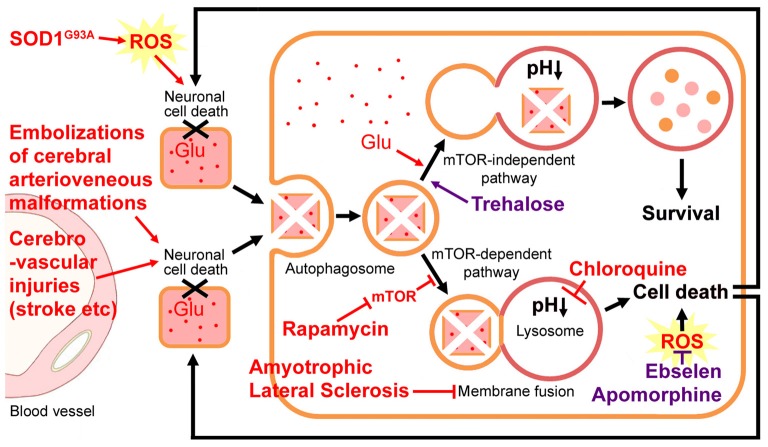
Hypothetical neuronal cell death-incomplete autophagy positive-feedback loop. Cerebrovascular injuries (such as multiple embolization sessions and strokes) induce neuronal cell death and subsequently autophagy occurs at the adjacent cells. Amyotrophic lateral sclerosis (ALS) impairs autophagosome-lysosome fusion and leads to magnified motor neuron cell death. Such a neuronal cell death-incomplete autophagy positive-feedback loop may be the key pathogenesis of ALS. Glutamate (Glu) accumulation, a mutation in superoxide dismutase (SOD), or reactive oxygen species (ROS) burst may promote this cell-death loop. Chloroquine represses autophagy through neutralizing lysosomal acidic pH, which is required for the activation of lysosomal hydrolase. Rapamycin activates autophagy by inhibiting the mammalian target of rapamycin (mTOR) kinase and exacerbates the motor neuron loss and exaggerates ALS progression. The mTOR-independent autophagy inducer trehalose is able to rescue the impaired fusion step and improve the disease course. With the different steps leading to the fusion of autophagosomes and lysosomes, the roles of rapamycin and trehalose may be detrimental and beneficial, respectively. Ebselen and Apomorphine are two antioxidants.

A defect in autophagosome-lysosome fusion has been observed in the ALS mouse model (Zhang et al., 2014). This lysosomal fusion deficiency may contribute to motor neuron degeneration (Zhang et al., 2014). Different from the mTOR-dependent autophagy, the mTOR-independent autophagy inducer trehalose is able to attenuate the lysosomal fusion deficiency and improve motor neuron functions in the SOD1^G93A^ ALS-model mice. Trehalose treatment significantly delayed disease onset, although it did not affect disease duration (the time course from ALS onset to death; Zhang et al., 2014). With different steps leading to the fusion of autophagosomes and lysosomes, the roles of rapamycin and trehalose may be detrimental and beneficial, respectively. The up-regulation of autophagosomes by rapamycin may induce early-to-intermediate autophagosome aggregation and subsequent cell death if the lysosomal fusion step is inhibited (Figure 1). While trehalose rescues the impaired fusion step, it results in aggregated autophagic degradation of the mutant SOD1 protein (Zhang et al., 2014).

Castillo et al. (2013) also found that trehalose led to autophagic degradation of the mutant SOD1 protein in NSC34 motor neuron cells, and it protected primary motor neurons against the excitotoxicity in mutant SOD1 transgenic astrocytes. Besides ALS, an inhibition of the autophagy-lysosome degradative pathway was also observed in a mouse model of human tauopathy (a severe neurodegenerative disorder caused by the accumulation of the tau protein). Interestingly, autophagy stimulation by trehalose reduces tau aggregates and improves neuron survival in the brainstem and the cerebral cortex (Schaeffer et al., 2012), which confirms the neuroprotective effects of trehalose on such neurodegenerative disorders. Thus, trehalose may be a prophylactic drug against the onset of ALS for patients with cerebrovascular injuries.

## Glutamate and ROS also Participate in Autophagy

On the other hand, neuronal cell death decreases neuronal glutamate (Glu) uptake, which is required for the rapid removal of Glu from the extracellular space, thus terminating the excitatory signal and reducing the excitotoxic neuronal damages (Heath and Shaw, 2002; Geevasinga et al., 2016). Glutamate itself also induces autophagy via nicotinic acid adenine dinucleotide phosphate (NAADP; a second messenger for glutamate) and lysosomal TPC (Ca^2+^-permeable two-pore channels), while mTOR activity was not affected by the glutamate treatment (Pereira et al., 2017). Therefore, glutamate may promote this neuronal cell death-autophagy positive-feedback loop (Figure 1).

Produced during cerebrovascular injuries, the reactive oxygen species (ROS) burst also participates in neuronal cell death and the autophagy process (Tang et al., 2016). The mutation in SOD1 accounts for approximate 20% of familial ALS (Kirby et al., 2005). A large number of studies with SOD1^G93A^ ALS-model mice provide strong evidence for the role of oxidative stress in the disease pathogenesis (Geevasinga et al., 2016; Spalloni and Longone, 2016). Additionally, ROS-mediated mitochondrial dysfunction, in connection with glutamate excitotoxicity, has been implicated in ALS pathogenesis (Xu et al., 2004). Glutamate excitotoxicity leads to excessive Ca^2+^ accumulation in mitochondria, resulting in the production of ROS that are toxic to neuronal cellular nucleotides and proteins. The mitochondria remain sensitive to oxidative damages, resulting in further mitochondrial dysfunction (Spalloni and Longone, 2016). The mitochondrial dysfunction, in turn, enhances glutamate-mediated excitotoxicity by disrupting the normal voltage-dependent Mg^2+^-mediated blockade of glutamate receptors (Bowling and Beal, 1995; Xu et al., 2004). Therefore, therapies targeting oxidative stress have also been highlighted as demonstrating great promise in slowing the progression of the disease (Figure 1). Recently, the FDA has granted orphan drug status to Edaravone (an antioxidant) for the treatment of ALS (Nagase et al., 2016).

## Neuron-Astrocyte Glutamate-Glutamine Cycle

**Figure 2:**
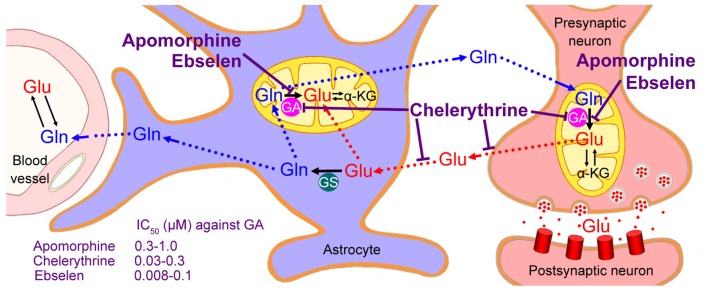
Neuron-astrocyte glutamate-glutamine cycle. Glutamate (Glu) released from the neurons is transported into astrocytes, converted to glutamine (Gln), and subsequently returned to the neurons and converted to glutamate by the mitochondrial enzyme glutaminase (GA). Only astrocytes (not neurons) express glutamine synthetase (GS). Although a small part of neuronal glutamate is synthesized from α-ketoglutarate (α-KG), most of it is converted from glutamine by the neuronal GA. Therefore, GA inhibitors, such as Ebselen, Chelerythrine and Apomorphine, might decrease neuronal glutamate levels and retard the progression of ALS; however, Chelerythrine suppresses not only neuronal glutamate synthesis but also glutamate/aspartate transporters, thus showing some excitotoxic effects.

Glutamate cannot permeate the blood-brain barrier easily so that it can accumulate in the brain. The glutamate-glutamine (Glu-Gln) cycle is the key pathway that regulates glutamate stores in neurons (Sonnewald and Schousboe, 2016). During excitatory neurotransmission, not all glutamate released from pre-synaptic neurons is recovered. Under normal conditions, excess glutamate not involved in transmission is taken up by astrocytes, which express high levels of the glutamate-specific transporters. Astrocytes specifically express the microsomal enzyme glutamine synthetase (GS), which catalyzes glutamate-to-glutamine conversion through ATP-dependent amidation. Glutamine is a non-neuroactive amino acid that can be released to extracellular fluids for subsequent uptake by neurons or transport to the blood vessel. Glutamine deamidation by mitochondrial glutaminase (GA) regenerates glutamate, closing the Glu-Gln cycle (Figure 2; Sonnewald and Schousboe, 2016).

This “glutamate-glutamine cycle” has been discovered several decades ago based on experiments using radio-labeled glutamate. The cycle has been confirmed by the fact that two distinct pools of glutamate and glutamine have been found. GS was expressed in astrocyte cells but not in neuron cells, which forms the basis of the idea that a cycle must exist in which glutamate released from the neurons is transported into astrocytes and converted into glutamine. The astrocytic glutamine is then returned to the neurons and converted into glutamate by GA, which has significantly higher activity in neurons than in astrocytes (Sonnewald and Schousboe, 2016). Cycling of glutamate and glutamine between astrocytes and neurons supports the provision of high concentrations of glutamate in neurons, but prevents excitotoxicity in the synaptic space (Conway and Hutson, 2016).

## Neuroprotective Roles of Glutaminase Inhibitors

Although biosynthesis via the tricarboxylic acid cycle (TCA) also contributes to the accumulation of presynaptic glutamate (about 15%–43% of glutamate is formed from α-ketoglutarate (α-KG) via the TCA cycle), a major part (usually >80%) of neuronal glutamate is converted from glutamine by the enzyme GA (McKenna et al., 1996). Thus, inhibition to GA might decrease neuronal glutamate to a non-toxic level and retard the progression of ALS (Figure 2).

Mammalian GA proteins are encoded by two paralogous genes, *Gls* and *Gls2*, both of which have been found in neurons (Márquez et al., 2016). Some small-molecule inhibitors against both types of GA were proven to be effective at preventing glutamate accumulation in human immunodeficiency virus (HIV)-infected macrophages (Zhao et al., 2004; Erdmann et al., 2007). The GA inhibitor 6-diazo-5-oxo-L-norleucine (DON) decreases glutamate released from activated microglia and rescues hippocampal neuron cell death after transient brain ischemia (Takeuchi et al., 2008; Shijie et al., 2009). Besides these prototypical GA inhibitors, like DON and bis-2-(5-phenylacetimido-1,2,4-thiadiazol-2-yl)ethyl sulfide (Shukla et al., 2012), three high-affinity GA inhibitors against both GLS and GLS2 have been recently identified: Apomorphine, Chelerythrine and Ebselen (Thomas et al., 2013).

Apomorphine is a dopamine agonist that has been used to treat Parkinson’s disease (Nomoto et al., 2015). A recent study demonstrated that Apomorphine attenuates motor dysfunction significantly in the SOD1^G93A^ transgenic mouse (Mead et al., 2013). A significant decline in rotarod performance was found in the ALS model mouse from postnatal day 40 (p40). However, this initial decline in motor neuron function was apparently delayed by Apomorphine treatment. At later stages of ALS progression, gait analysis showed a significant delay in the decline of both fore-limb and hind-limb stride length after Apomorphine treatment. These treatments also reduced oxidative damages and improved survival following oxidative insult in fibroblast cells from ALS patients (Mead et al., 2013). Therefore, the authors attribute the neuroprotective effect to its antioxidant properties. However, in the above study of the ALS mouse model, 5 mg/kg of Apomorphine was administrated daily. This is equal to 0.55 mg/kg for humans (about 33 mg for a man daily), which is much higher than the maintenance dose of 2–6 mg per day (Nomoto et al., 2015). On the other hand, the C_max_ of Apomorphine at the effective dose for the treatment of ALS was presumed to be about 20 ng/mL (0.075 μM; Mead et al., 2013). However, the average IC_50_ of Apomorphine against GA is much higher (0.3–1.0 μM; Thomas et al., 2013). Furthermore, the adverse side effects of Apomorphine administration have been widely reported (Bhidayasiri et al., 2016). Thus, it should not be used at high dosages.

Chelerythrine suppresses not only neuronal glutamate synthesis but also glutamate/aspartate transporters (Bull and Barnett, 2002). Failure or reversal of the glutamate transport system causes an elevation of extracellular glutamate and contributes to the onset of excitotoxic neuronal damages (Bull and Barnett, 2002; Sonnewald and Schousboe, 2016). Correspondingly, although a reduction in total retinal glutamate levels following Chelerythrine treatment was observed, the extracellular glutamate was accumulated in the synaptic space (Bull and Barnett, 2002). Chelerythrine treatment resulted in the significant swelling of the retinal inner plexiform layer and a significant thinning of the inner nuclear layer, indicating neuronal cell death in the retina (Bull and Barnett, 2002). Thus, Chelerythrine may not be an ideal drug for ALS.

Ebselen is a synthetic organoselenium drug with high anti-inflammatory, antioxidant and cytoprotective activities (Parnham and Sies, 2013). It works as a mimic of glutathione peroxidase and it can also react with peroxynitrite (Parnham and Sies, 2013). Therefore, Ebselen may prevent neuronal cell death caused by cerebrovascular injuries or SOD mutations, as mentioned above (Figure 1). Although no direct effect of Ebselen on ALS has been reported up to now, it has been extensively used in a variety of experimental animal models of cerebrovascular injuries and used as a neuroprotectant in treatments for patients following acute ischemic stroke and aneurysmal subarachnoid hemorrhage (Saito et al., 1998; Yamaguchi et al., 1998; Ogawa et al., 1999; Koizumi et al., 2011; Parnham and Sies, 2013; Singh et al., 2016). For GA inhibition, its average IC_50_ is 0.008–0.1 μM (Thomas et al., 2013). Normal doses (such as 3 × 200 mg capsules, 1–2 times a day) meet this requirement. Ebselen is a blood-brain barrier penetrant drug and it has been tested clinically in some diseases, thus enabling us to recommend it for the treatment of ALS.

## Conclusions and Future Prospects

Here, we hypothetically proposed a correlation between cerebrovascular injury-induced cell death and autophagic flux defect in ALS patients. Nevertheless, direct experimental evidence is still lacking. The impairment in autophagosome-lysosome fusion can be mimicked by bafilomycin A1 treatment (Ejlerskov et al., 2013), so if ALS onset was observed in the bafilomycin-A1-treated stroke model mice (Chen et al., 2015) at the convalescent period, these correlations would be confirmed. Although the autophagosome-lysosome fusion defect has been found in the ALS mouse model (Zhang et al., 2014), direct evidence for the relevance of the cellular components required for the fusion is still missing. More accurate molecular biological studies are required to test this hypothesis.

Therapeutic effects of trehalose for ALS patients also need clinical confirmation. If human clinical trials succeed, it might be considered to be a prophylactic drug for ALS for high-risk populations, such as convalescent patients of stroke and some AVM patients with significant perinidal angiogenesis, who just received embolization sessions. In mouse experiments, 2% trehalose containing water has been adopted for the SOD1^G93A^ mouse by *ad libitum* consumption (Schaeffer et al., 2012; Zhang et al., 2014). Whether similar trehalose treatments should be given to humans needs further investigation. There is no recommendation from the FDA or the Center for Sciences in the Public Interest on this sugar. It would be best to follow the World Health Organization (WHO) guidelines and restrict the intake of all sugars, including trehalose to 50 g per day (Richards et al., 2002). For the typical person, the daily potable water quantity is about 1500–2500 ml, so the use of 2% trehalose means keeping to 30–50 g per day. Thus, the WHO guideline may be a feasible one. Nevertheless, a large part of trehalose sugar would be broken down into glucose by trehalase on the intestinal mucosa (Richards et al., 2002), and therefore oral administration may not be a very effective method. Thus, trehalose injection might be an alternative option (Echigo et al., 2012).

Ebselen is well tolerated and shows good effects on emotional processing. Thus far it has progressed to phase III clinical trials for bipolar disorder (Parnham and Sies, 2013; Singh et al., 2016). No toxicity or side effects have been reported so far for Ebselen at the administered doses. Its IC_50_ of GA inhibition is very low and its antioxidant capacity is very strong. Thus, it might be a promising therapeutic drug for the treatment of ALS. To retard (or even stop) ALS progression, trehalose and Ebselen might be adopted in combination, considering that they disrupt the neuronal cell death-incomplete autophagy positive-feedback loop and the neuronal glutamate accumulation cycle, respectively.

## Author Contributions

SY conducted the literature search and drafted the manuscript. Z-WZ contributed to the discussion of ideas and helped with the writing. Z-LL contributed to the discussion of ideas and helped with the writing.

## Conflict of Interest Statement

The authors declare that the research was conducted in the absence of any commercial or financial relationships that could be construed as a potential conflict of interest.
